# Development of raster scanning IMRT using a robotic radiosurgery system

**DOI:** 10.1093/jrr/rraa136

**Published:** 2021-01-18

**Authors:** Hiroya Shiomi, Yuichi Akino, Iori Sumida, Norihisa Masai, Ryoong-Jin Oh, Kazuhiko Ogawa

**Affiliations:** Miyakojima IGRT Clinic, Miyakojima-ku, Osaka 534-0021, Japan; Department of Radiation Oncology, Osaka University Graduate School of Medicine, Suita, Osaka 565-0871, Japan; Soseikai Clinic CyberKnife Center, Fushimi, Kyoto 612-8248, Japan; RADLab Inc., Kawachinagano, Osaka 586-0092, Japan; Soseikai Clinic CyberKnife Center, Fushimi, Kyoto 612-8248, Japan; Oncology Center, Osaka University Hospital, Suita, Osaka 565-0871, Japan; Department of Radiation Oncology, Osaka University Graduate School of Medicine, Suita, Osaka 565-0871, Japan; Department of Radiology, North Medical Center Kyoto Prefectural University of Medicine, Yosano-cho, Yosa-gun, Kyoto 629-2261, Japan; Miyakojima IGRT Clinic, Miyakojima-ku, Osaka 534-0021, Japan; Department of Radiation Oncology, Osaka University Graduate School of Medicine, Suita, Osaka 565-0871, Japan

**Keywords:** CyberKnife, stereotactic body radiotherapy, intensity-modulated radiotherapy, prostate cancer

## Abstract

Treatment time with the CyberKnife frameless radiosurgery system is prolonged due to the motion of the robotic arm. We have developed a novel scanning irradiation method to reduce treatment time. We generated treatment plans mimicking eight-field intensity-modulated radiotherapy (IMRT) plans generated for the Novalis radiosurgery system. 2D dose planes were generated with multiple static beam spots collimated by a fixed circular cone. The weights of the uniformly distributed beam spots in each dose plane were optimized using the attraction–repulsion model. The beam spots were converted to the scanning speed to generate the raster scanning plan. To shorten treatment time, we also developed a hybrid scanning method which combines static beams with larger cone sizes and the raster scanning method. Differences between the Novalis and the scanning plan’s dose planes were evaluated with the criterion of a 5% dose difference. The mean passing rates of three cases were > 85% for cone sizes ≤ 12.5 mm. Although the total monitor units (MU) increased for smaller cone sizes in an inverse-square manner, the hybrid scanning method greatly reduced the total MU, while maintaining dose distributions comparable to those with the Novalis plan. The estimated treatment time of the hybrid scanning with a 12.5 mm cone size was on average 22% shorter than that of the sequential plans. This technique will be useful in allowing the CyberKnife with conventional circular cones to achieve excellent dose distribution with a shortened treatment time.

## INTRODUCTION

CyberKnife (Accuray Inc., Sunnyvale, CA, USA) is a frameless image-guided robotic radiosurgery system that consists of a linear accelerator mounted on a robotic arm [1]. This system enables highly conformal dose distribution by irradiation from multiple non-coplanar beam angles. In addition, the motion of the robotic arm coupled with the Synchrony system (Accuray) enables motion tracking radiotherapy to targets affected by respiratory motion, such as lung and liver tumors [2, 3]. The target localization system (TLS), which consists of two orthogonal kilovoltage X-ray imaging systems, can detect patient translation and rotation by detecting skull, spine or fiducial markers implanted in the patient. The IRIS collimator (Accuray) enables dynamic adjustment of collimator size and reduces monitor units (MU) [4]. The CyberKnife has been used for various extracranial lesions such as lung, liver and prostate [5–7].

Although the CyberKnife has the important potential of achieving excellent dose distributions, treatment time is extended by the motion of the robotic arm. Recently, the InCise (Accuray) multi-leaf collimator (MLC) became available [8–10]. The MLC can modulate the beam intensity inside each beam, achieving comparable dose distributions with fewer beam angles and a shortened treatment time. However, the InCise can only be mounted on the CyberKnife M6 (Accuray), and many institutions with older versions or financial considerations still use circular collimators for stereotactic body radiotherapy (SBRT).

The purpose of this study was to overcome following two problems of conventional CyberKnife treatment: (i) multiple beams irradiated from many beam angles (nodes) take a long time due to the motion of the arm, resulting in an extended total treatment time; and (ii) the dose distributions of the CyberKnife sequential treatment plans are usually not homogeneous compared to those with linac-based intensity-modulated radiotherapy (IMRT) plans [11–15]. The rapid, flexible and precise targeting provided by the robot arm and gantry head of the CyberKnife system has major potential in the application of motion tracking irradiation: if the CyberKnife head moved continuously during irradiation, it would be able to generate a photon fluence similar to IMRT beams established by a linear accelerator with MLC. Although prostate cancer treatments by CyberKnife often require multiple (>50) beams, IMRT-like irradiation may achieve homogeneous dose distribution with many fewer beam nodes.

We recently developed the concept of a scanning irradiation technique which shortens treatment time. Here, the feasibility of the technique was evaluated by generating prostate SBRT plans that mimicked clinical IMRT plans generated for the Novalis radiosurgery system (BrainLAB, Munich, Germany).

## MATERIALS AND METHODS

### Treatment plans

**Fig. 1.**
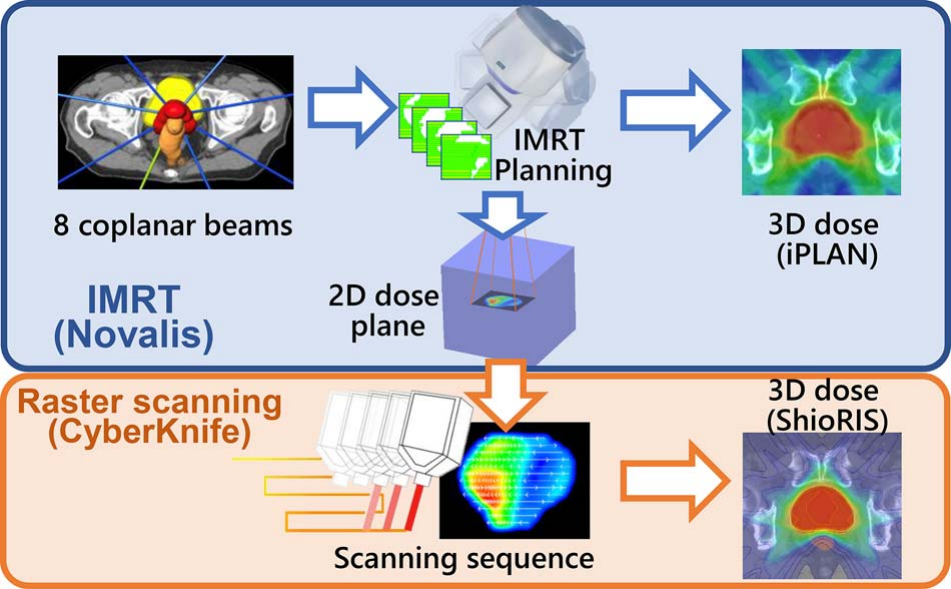
Schematic image of generation of a raster scanning plan from 2D dose planes derived from a standard MLC-based IMRT plan and calculation of 3D dose distribution of the patient.

Figure 1 shows the scheme of this study. Because our current system cannot optimize photon fluence for IMRT planning, an iPLAN Dose version 4.1.2 (BrainLAB), a commercial treatment planning system (TPS), was used as an optimizer of photon fluence. First, an IMRT plan was generated for the Novalis. Subsequently, scanning sequences for CyberKnife were optimized by mimicking the photon fluence of the IMRT plan.

Following approval of the institutional review board, we analyzed the treatment plans of three patients who received prostate IMRT as a reference. Volumes of the target and critical organs are listed in supplementary Table 1, see online supplementary material. The treatment plans were generated with the iPLAN TPS with a 2-mm grid size, and the treatments were conducted using a Novalis radiosurgery system with an M3 micro-MLC (BrainLAB). Eight coplanar beams with 6 MV photon beam energy were used. The prescribed dose was 72 Gy/30 fractions. The planning target volume (PTV) margin was 3 mm for the posterior of the prostate and 5 mm for other directions. The prescribed dose in this study was 36.25 Gy/5 fractions, and the dose of the reference plan was rescaled. The doses were normalized at PTV *D*_95%_ (dose covering 95% of PTV). The iPLAN TPS generated a 2D dose plane of each treatment beam calculated at the isocenter plane with a 10 cm depth in the water-equivalent material for quality assurance. The dose planes were used as the reference of the raster scanning sequences instead of optimizing the fluence maps.

### Raster scanning

**Fig. 2.**
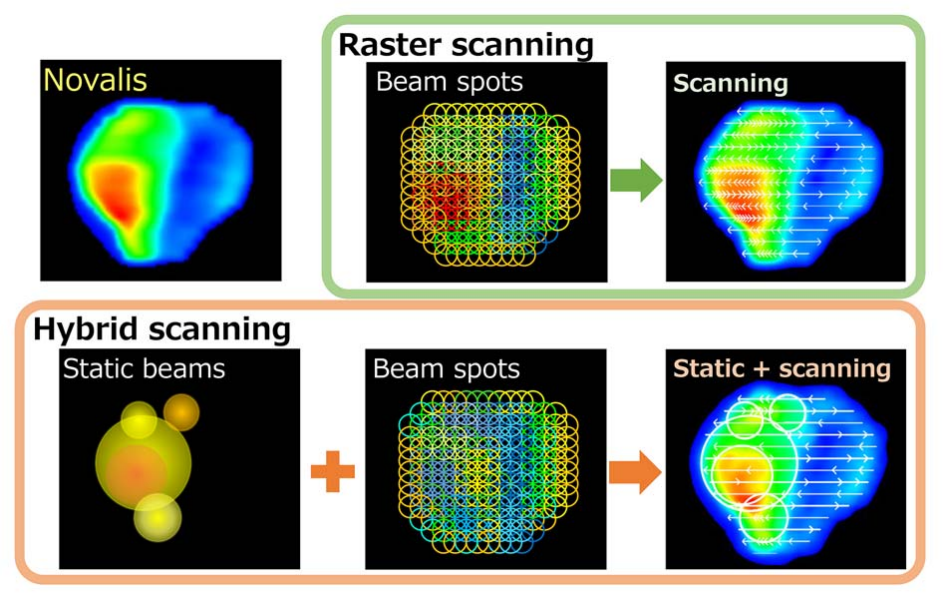
Scheme of the algorithm for generating the raster scanning and hybrid scanning sequences.

As illustrated in the bottom row of Fig. 1, the raster scanning uses the circular beam of the CyberKnife. The photon beam is continuously irradiated with movement of the head of the CyberKnife to shape the radiation field to the target. Although the dose rate is assumed to be constant, the speed of the scanning motion is adjusted to modulate the intensity inside the treatment field. Figure 2 shows the scheme of the raster scanning and hybrid scanning irradiation methods generated from the 2D dose plane of the IMRT plan for the Novalis. For the raster scanning method, beams are irradiated with a single fixed cone-type collimator. In this study, we evaluated cone sizes ranging from 5 to 25 mm φ. First, equally distributed static beams were considered. The dose plane was calculated at an 80 cm source-to-axis distance and 10 cm depth. Optimization was performed using an attraction–repulsion model (ARM). The ARM was originally developed for optimization of low-dose-rate (LDR) interstitial brachytherapy for prostate cancer [16], and subsequently extended to high-dose-rate (HDR) brachytherapy [17]. This model is based on Gauss’ law regarding the distribution of charged particles in an electric field. The charged particles representing LDR iodine seeds or the dwell time of an HDR iridium source at each dwell position are affected by the Coulomb forces from the electric field. All pixels or voxels of the uniformly distributed optimization grid generate attraction or repulsion, representing an increase or decrease in dose (or dose rate), respectively. In this study, the ARM algorithm was extended to optimize the weights of the CyberKnife spot beams. The attraction force increased the beam weights at the control points around the pixel, whereas the repulsion force decreased these weights. The grid size for optimization using ARM was 1 mm for horizontal and vertical axes. The weight of each static beam was optimized by minimizing the objective function, calculated as the difference between the dose planes of the Novalis plan and raster scanning.

After optimization, the static beams were converted to continuously moving beams with consideration to the beam weight at each point. The spacing of control points for the continuously moving beams was one-half and one-quarter of the cone diameter for cone sizes ≤12.5 and ≥15 mm, respectively. The dose rate was assumed to remain constant (800 MU/min for a CyberKnife G4). In the right figure of raster scanning in Fig. 2, arrows and their intervals represent the direction and speed of scanning, respectively.

To evaluate the dose–volume-histogram (DVH), the 3D dose distribution of the patient was calculated using the ShioRIS 2.0 software (RADLab, Inc.). The resolution of dose calculation was the same as that of computed tomography (CT) images. The dose was calculated with a pencil beam dose calculation algorithm as follows:

$$\begin{equation*} {\mathrm{Dose}}_{(\varPhi, d,x,\mathrm{SSD})}\,{=}\,\mathrm{MU}\cdot{((\mathrm{SSD}\,{+}\,d)/800)}^{-2}\cdot{TPR}_{(\varPhi, d)}\cdot{OAR}_{(\varPhi, d,x)}\cdot{OPF}_{(\varPhi )} \end{equation*}$$

where *Φ*, *d*, *x* and SSD represent the nominal cone diameter, depth from the body surface, offset distance from the central axis of the beam and source-to-surface distance, respectively. TPR, OAR and OPF represent the tissue-phantom-ratio, off-axis-ratio and output factor, respectively; these data were derived from the beam data of a CyberKnife G4 system. Heterogeneity corrections were considered only for the primary beam attenuation by using the equivalent path length for calculation of TPR. Dose calculation accuracy of the ShioRIS software has been reported elsewhere [18].

To further reduce the beam-on time, we also developed a hybrid scanning technique which combines the raster scanning technique with several static beams with larger cone sizes. First, a copy dose plane of the Novalis plan was generated. Within the copied dose plane, an area which can include the 60-mm φ beam is sought. If an area is found, the dose of the 60-mm φ cone is subtracted from the copied dose plane with a maximum weight which does not exceed the copied dose plane. This process is performed for the entire map and then repeated with a smaller cone size. The cone sizes for static beams were 20–60 mm φ. Finally, the raster scanning technique is used to generate the fluence mimicking the remaining dose plane. In this study, we evaluated the 10 and 12.5-mm cone sizes for raster scanning.

### Standard CyberKnife planning

For reference, sequential CyberKnife treatment plans were also generated using a MultiPlan TPS (version 4.6.0, Accuray, Inc.). The plans were generated with the Iris collimator, and the doses were calculated with a pencil beam algorithm with ‘high resolution. An example of the optimization parameters is listed in supplementary Table 2, see online supplementary material. The parameters of 40 min and 50 MU were used for time reduction and beam reduction functions, respectively. The number of nodes were 55, 62 and 60 for cases 1, 2 and 3, respectively.

### Evaluations

To evaluate how much the techniques proposed in this study reproduce the dose planes of the Novalis plan, dose difference and gamma analysis were evaluated using the Akilles RT software (RADLab, Inc., Osaka, Japan) [19]. Points with a dose <10% of the maximum dose were ignored.

To evaluate the homogeneity of the PTV dose, the homogeneity index (HI) was calculated using following formula:

$$ \mathrm{HI}=\frac{D_{5\%}-{D}_{95\%}}{D_p} $$

where *D*_5%_ and *D*_95%_ represent the minimum dose in 5 and 95% of the PTV, and *D*_p_ represents the prescribed dose. For rectum and bladder, the generalized equivalent uniform dose (gEUD) [20] was calculated:

$$ \mathrm{gEUD}={\left(\sum_i\frac{V_i}{V}{D_i}^{1/n}\right)}^n $$

where *n* is a parameter that describes the volumetric dependence of the dose–response relationship for each organ. The values of *n* were 0.12 and 0.5 for rectum and bladder, respectively, as previously reported by Burman *et al.* [21].

## RESULTS

**Fig. 3.**
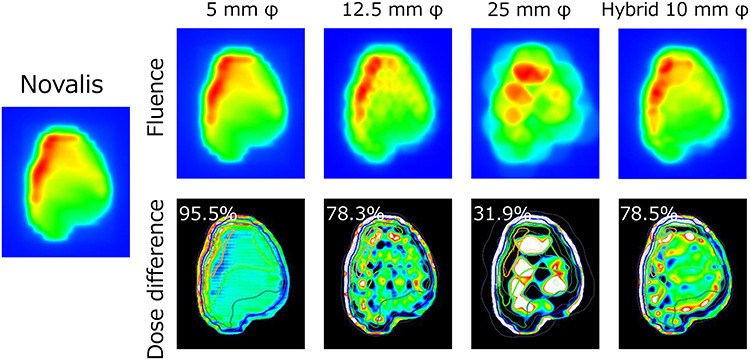
Upper row: dose planes calculated for raster scanning with 5-, 12.5- and 25-mm φ cone sizes and hybrid scanning with a 10-mm φ cone size for scanning. Lower row: dose difference calculated between the dose planes of the proposed methods and those of the Novalis plan with a criterion of 5%. White and black regions inside the colored region represent values >5% and values <−5%, respectively. Percentage values on each figure represent the pass rate calculated for an area ≥10% of the maximum value of each dose plane.

Figure 3 shows an example of the dose planes of the Novalis plan exported from the iPLAN TPS and the dose plane generated with the raster scanning method using 5-, 12.5- and 25-mm φ cones. Dose difference showed that the 5-mm φ cone accurately reproduced the dose plane of the Novalis plan within the criteria of 5%, whereas the 25-mm φ cone showed a deteriorated dose plane.

Beam-on time was calculated from total MU and the dose rate of 800 MU/min. For hybrid scanning plans with 10 and 12.5-mm φ cone sizes, the mean of the total beam-on times were 28.5 and 21.7 min, respectively. These were significantly shorter than plans of raster scanning with the same cone diameters. Whereas the sequential plans used 55, 62 and 60 nodes, the raster scanning and hybrid scanning plans used only eight beams. A shorter total treatment time would therefore be expected using the hybrid scanning method. The beam-on times of the sequential plans calculated for 800 MU/min were 5.2, 5.0 and 5.0 min for cases 1, 2 and 3, respectively. The mean time needed for each arm motion was 0.47 min. Although this is a very rough calculation and the value strongly depends on the individual plan, the raster scanning and hybrid scanning plans with eight coplanar beams would take only 3.7 min for arm motions. Total treatment time was estimated by adding the time needed for arm motion to the beam-on time. Details of the MU and beam-on time are listed in supplementary Table 3, see online supplementary material.

**Fig. 4.**
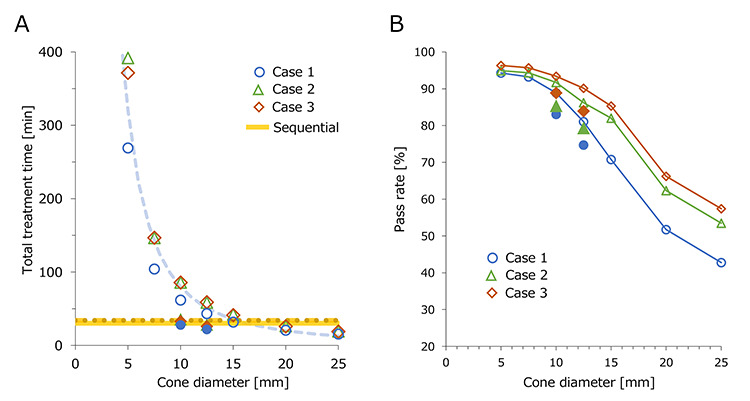
(**A**) Estimated total treatment time plotted against cone diameter. The blue dashed line represents an approximation curve of the inverse square. For the CyberKnife sequential plan, the solid, dashed and dotted lines represent case 1, 2 and 3, respectively. (**B**) Pass rate of the dose difference calculated with a criterion of 5% plotted against cone diameter. The mean of eight beams is shown. Frame- and filled-plots represent the raster scanning and hybrid scanning, respectively.

In Fig. 4A, the total treatment time of each plan was plotted against the cone diameter. With smaller cone sizes, total treatment time was greatly increased. The dashed line represents an approximation formula:

$$ T=8000/{\upvarphi}^2 $$

where *T* and φ represent the total treatment time and cone diameter, respectively. This curve showed a good fit with the results of the raster scanning, indicating that the treatment time of the raster scanning correlated with the inverse square of the cone diameter. The estimated total treatment time of the hybrid scanning method using a 12.5-mm cone size was 21.7, 28.5 and 25.9 min for cases 1, 2 and 3, respectively. The estimated treatment time was on average 22% shorter than the sequential plans.

Figure 4B shows the mean dose difference values of the eight beams calculated for the raster scanning and hybrid scanning methods. The pass rate values were plotted against the cone sizes, showing a decrease in values with the larger cone diameter. Although cases 2 and 3 showed similar pass rates, case 1 showed a lower pass rate. The reproducibility of the fluence maps would depend on the complexity and modulation of the plans. The pass rates of hybrid scanning were slightly lower than those of the raster scanning method. The values represent the similarity of the fluence.

**Fig. 5.**
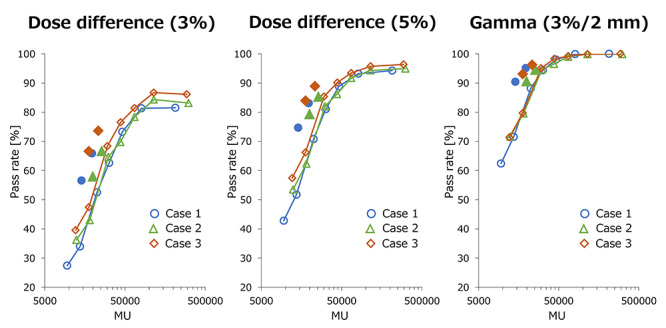
Dose difference and gamma pass rate plotted against total MU. The horizontal axis is the log scale. Frame- and filled-plots represent raster scanning and hybrid scanning, respectively.

In Fig. 5, the dose difference and the mean gamma pass rate between the dose planes generated for each plan and extracted from iPLAN TPS were plotted against the total MU. Only the horizontal axis is a log scale. The raster scanning showed that the pass rates of the dose difference and gamma analysis were improved with a larger MU. Interestingly, the hybrid scanning method significantly improved the values with much smaller MUs than those of the raster scanning method. This is likely because a large number of MUs were delivered with large cone sizes. Details of the passing rate values of dose difference calculated with the criterion of 5% are shown in supplementary Table 4, see online supplementary material. Mean travel lengths of the eight beams were 177.5 and 35.6 cm and maximum velocities were 0.10 and 0.48 cm/s for the 5 and 25-mm cone sizes, respectively. According to the vendor, the maximum velocities of the robotic arm are 3 and 10 cm/s for treatment and other motions, respectively. Even when using the largest cone size and small fractional doses, such as 2 Gy/fraction, the velocity of the arm motion will be within the machine specification.

**Fig. 6.**
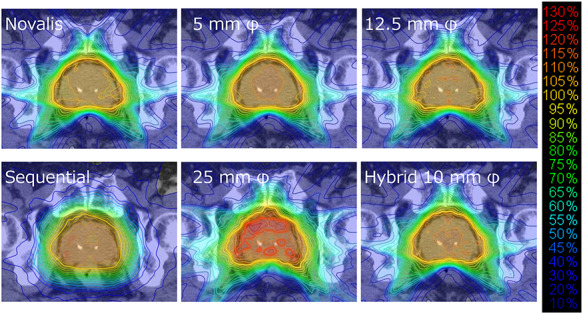
Transverse dose distribution of the Novalis plan, CyberKnife sequential plan and plans generated with raster scanning methods.

**Fig. 7.**
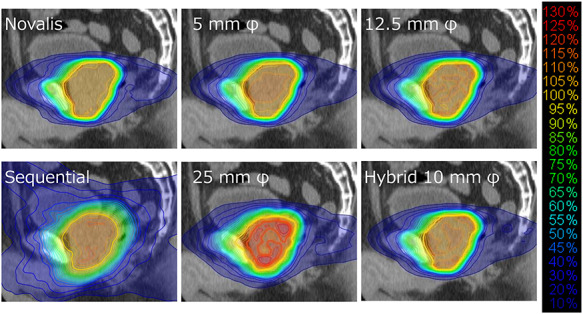
Sagittal dose distributions of the Novalis plan, CyberKnife sequential plan and plans generated with raster scanning methods.

We also calculated the 3D dose distributions of the raster scanning and hybrid scanning plans. Figures 6 and 7 show the transverse and sagittal dose distributions of the Novalis and raster scanning plans, respectively. The Novalis plan showed uniform dose distribution inside the prostate. Although the raster scanning plan with a 5-mm φ cone size also showed uniform dose distribution, hot spots increased with larger cone sizes. The hybrid scanning plan with a 10-mm φ cone size also demonstrated uniform dose distribution, which showed similar homogeneity to the plan of the 12.5-mm φ raster scanning.

**Fig. 8.**
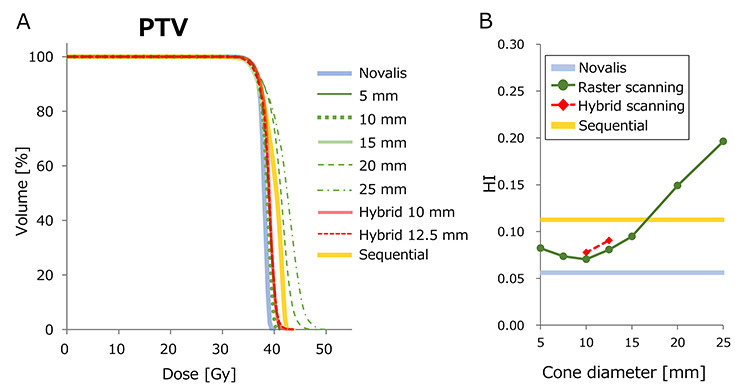
(**A**) DVH curves of PTV of case 1. (**B**) HI of each plan plotted against cone diameter.

**Fig. 9.**
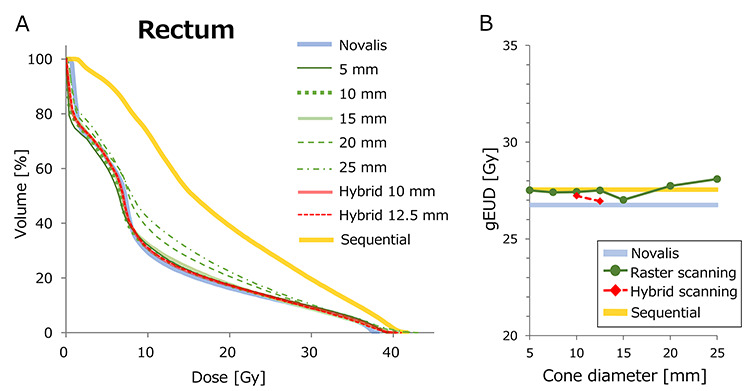
(**A**) DVH curves of rectum of case 1. (**B**) gEUD of each plan plotted against cone diameter.

**Fig. 10.**
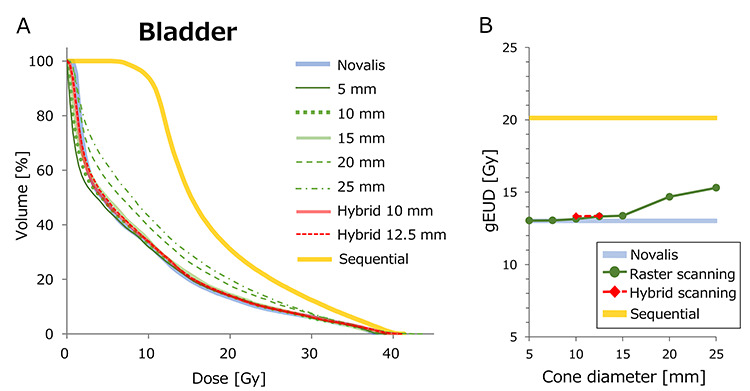
(**A**) DVH curves of bladder of case 1. (**B**) gEUD of each plan plotted against cone diameter.

The DVH results of case 1 are demonstrated in Figs 8–10. Figure 8A shows the DVH of PTV calculated for various plans. The Novalis plan showed the steepest DVH curve, representing the most homogeneous dose distribution. Figure 8B shows the HI values plotted against the cone diameter. Although cone sizes ≥20 mm showed inhomogeneous dose distributions, the plans of cones ≤15 mm showed better homogeneity. The hybrid plans with 10 and 12.5 mm φ also showed homogeneous dose delivery to the PTV. Figure 9A shows the DVH of rectum. Although the plans with cones ≥20 mm showed a higher rectal dose than those of the Novalis plan, the plans with cones ≤15 mm showed a DVH closely similar to that of the Novalis plan. Figure 9B shows the gEUD of rectum plotted against cone diameter. Although both raster scanning and hybrid scanning showed a higher gEUD than the Novalis plan, the differences were within 2 Gy, even for raster scanning with cone sizes ≥20 mm. Because the parameter *n* for volumetric dependence was 0.12 for rectum, the calculation of gEUD strongly depends on a high dose. The DVH in the high dose region >40 Gy showed small variation among plans, resulting in the modest differences in gEUD. Although the CyberKnife sequential plan showed high doses especially in low-middle doses, the difference in gEUD between the Novalis and sequential plans was within 1 Gy. Figure 10A and B show the DVH and gEUD of bladder. The CyberKnife sequential plan showed the highest dose, probably due to the use of non-coplanar beams. Plans with cone sizes ≤15 mm showed closely similar DVH curves to that of the Novalis plan. The gEUD also showed differences of <1 Gy for cone sizes ≤15 mm φ. The increases in gEUD from the Novalis plan were 2.3 and 3.2 Gy for 20- and 25-mm φ cone sizes, respectively.

**Fig. 11.**
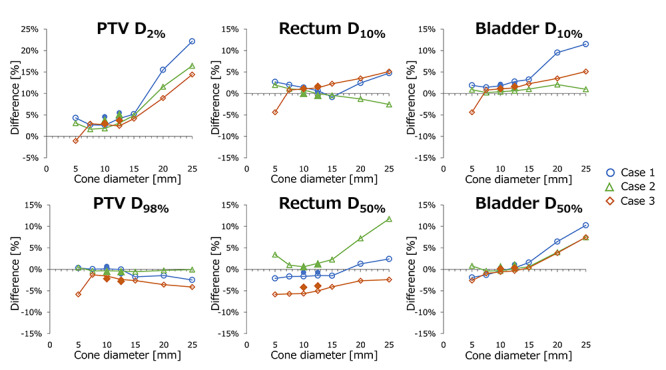
Dose–volume metrics of the raster scanning and hybrid scanning plans. The values from the Novalis plan are shown. Vertical axis represents percent prescribed dose. Frame- and filled-plots represent raster scanning and hybrid scanning, respectively.

The differences in the dose–volume metrics of the PTV, rectum and bladder from the values of the Novalis plan are shown in Fig. 11. Raster scanning with 20- and 25-mm φ cone sizes showed a significant increase in D_2%_, representing the near-maximum dose [22]. Because the dose was normalized at D_95%_, D_98%_, representing the near-minimum dose, showed modest variation. For rectal and bladder doses, the variations in the DVH metrics were small for ≤ 5 mm φ cone sizes.

## DISCUSSION

In this study, we developed novel irradiation methods for CyberKnife to shorten treatment time. Because the treatment plans were generated with only eight nodes, the time required for arm motion will be greatly reduced compared to standard CyberKnife treatment plans with multiple nodes. To evaluate the performance of the methods, we investigated how they reproduced the dose distribution of a clinical prostate case treated with the Novalis system. The fluence maps were generated using the iPLAN TPS instead of developing the fluence optimizer. Using small cone sizes, raster scanning generated dose plans comparable to those optimized by the iPLAN TPS. As shown in Figure 5, the dose differences were <90% for most plans. Perfectly identical fluences are not needed to achieve the goal of generating treatment plans with a shorter treatment time, target homogeneity and comparable OAR doses. For the cases evaluate in this study, overlap between the target and critical organs was not large. For cases with a large overlap region or having the small bowel close to the target, however, the accuracy of the generated fluence map would be more important because of the higher intensity modulation and steeper dose gradient these require.

Recently, VMAT has shown great potential for providing excellent dose distribution with very high throughput [23, 24]. Compared to VMAT, CyberKnife treatments take much longer because of the multiple robotic arm motions. Kearney *et al*. previously reported a simulation study of continuous arc irradiation using a CyberKnife M6 with InCise MLC [25]. Their technique achieved dose distributions comparable to those generated by step-and-shoot irradiation with a CyberKnife M6 but with a shorter treatment time. Tomida *et al*. previously reported that the estimated time of prostate cancer treatment using the InCise MLC was 32 ± 4 min, which was ~21% shorter than that using the Iris collimator [26]. To improve treatment throughput, such techniques are required. The raster-scanning method presented in this study showed a trade-off between plan quality and MU. Although the total MU can be reduced by use of a large cone size, plans generated with cone sizes ≥20 mm will not be clinically acceptable. The estimated treatment time of the plans using small cone sizes ≤15 mm was longer than that of sequential plans. Therefore, the raster scanning method will not be acceptable in clinical use. In contrast, the hybrid scanning method with a 10-mm cone size showed equivalent treatment time to the sequential plans, and the method with 12.5-mm cone size reduced treatment time by an average of 22%. For institutions which are not equipped with the InCise MLC, however, hybrid scanning with a 12.5-mm cone size will also be helpful in increasing treatment efficacy.

This study included the following limitations. First, the dose planes of the reference plan were optimized using an iPLAN TPS with beam data from the Novalis linear accelerator. The goal of this study was to investigate the feasibility of the raster scanning technique. This study did not include the optimization process in which intensity modulation is adjusted by iterative dose calculations and evaluations. The methods presented in this study can be expanded to non-coplanar beams. We also generated treatment plans with non-coplanar beams whose gantry angles were close to the beam orientations of the nodes of the CyberKnife. As illustrated in supplementary Figs 1–3, similar results were observed, even though beam angles from the patient’s posterior direction were limited. If the fluence maps were optimized with consideration of the scanning sequences of the non-coplanar beams, our methods would provide improved dose distributions. As shown in Figs 8B and9B, the smallest cone size did not always show the best target homogeneity or OAR dosed. Case 3 showed that the 5-mm cone showed minimum dose coverage of the PTV. The reference fluence maps of the Novalis plan were generated with the MLC-shaped segments calculated at a 10-cm depth in water-equivalent material. Although a small cone size successfully generated fluence maps which mimicked the reference maps, these were generated with circular cones. The similarity of the fluences at shallower and deeper depth would be worse than that at a 10-cm depth. This problem will be resolved with inclusion of the optimization process. For fluence map optimization, the spacing of the control points was one-half and one-quarter of the cone diameter for cones sized ≤12.5 and ≥15 mm, respectively. For the raster scanning plans with 5-mm cone size, the total number of circular beams needed to generate eight fluence maps were 6989, 10 125 and 9597, for patients 1, 2 and 3, respectively. If using smaller spacing for control points, the results would be improved, although the calculation time will also increase. Second, this study was virtually simulated, and current commercial treatment units cannot conduct scanning irradiation. However, the current version of the CyberKnife has the ability to achieving real-time motion tracking irradiation [3, 27]. Because the scanning speed was much slower than the maximum velocity of the arm motion, accurate beam delivery is expected. We consider that the CyberKnife has sufficient potential to allow the conduct of raster scanning irradiation, although the beam angles should be selected from available positions. Finally, the calculation of treatment time did not consider the intervals required for intra-node head adjustments, albeit that these would not take a long time. The total treatment time described above was roughly calculated. Nevertheless, the methods presented in this study have great potential in reducing the treatment time compared with the sequential method.

## CONCLUSION

A raster scan technique using the CyberKnife system successfully generated dose distributions comparable to those of linac-based IMRT. Because the CyberKnife system can move quickly in motion tracking mode, raster scan irradiation will be feasible, and treatment time will be considerably shortened compared to conventional CyberKnife plans. Effective beam delivery will be achieved when the method is combined with large static beams.

## Supplementary Material

Suppl_Figure_1_R5_rraa136

Suppl_Figure_2_R5_rraa136

Suppl_Figure_3_R5_rraa136

Supplementary_Tables_R4_rraa136

## References

[1] Antypas C, Pantelis E. Performance evaluation of a CyberKnife G4 image-guided robotic stereotactic radiosurgery system. Phys Med Biol 2008;53:4697–718. 18695294 10.1088/0031-9155/53/17/016

[2] Ricotti R, Seregni M, Ciardo D et al. Evaluation of target coverage and margins adequacy during CyberKnife lung optimized treatment. Med Phys 2018;45:1360–8. 29431863 10.1002/mp.12804

[3] Akino Y, Sumida I, Shiomi H et al. Evaluation of the accuracy of the CyberKnife synchrony respiratory tracking system using a plastic scintillator. Med Phys 2018;45:3506–15. 10.1002/mp.13028 29858498

[4] Echner GG, Kilby W, Lee M et al. The design, physical properties and clinical utility of an iris collimator for robotic radiosurgery. Phys Med Biol 2009;54:5359–80. 19687567 10.1088/0031-9155/54/18/001

[5] Iwata H, Ishikura S, Murai T et al. A phase I/II study on stereotactic body radiotherapy with real-time tumor tracking using CyberKnife based on the Monte Carlo algorithm for lung tumors. Int J Clin Oncol 2017;22:706–14. 28429140 10.1007/s10147-017-1123-0

[6] Su TS, Liang P, Liang J et al. Long-term survival analysis of stereotactic ablative radiotherapy versus liver resection for small hepatocellular carcinoma. Int J Radiat Oncol Biol Phys 2017;98:639–46. 28581406 10.1016/j.ijrobp.2017.02.095

[7] King CR, Brooks JD, Gill H et al. Stereotactic body radiotherapy for localized prostate cancer: Interim results of a prospective phase II clinical trial. Int J Radiat Oncol Biol Phys 2009;73:1043–8. 18755555 10.1016/j.ijrobp.2008.05.059

[8] Jin L, Price RA, Wang L et al. Dosimetric and delivery efficiency investigation for treating hepatic lesions with a MLC-equipped robotic radiosurgery-radiotherapy combined system. Med Phys 2016;43:727–33. 26843236 10.1118/1.4939259

[9] Jang SY, Lalonde R, Ozhasoglu C et al. Dosimetric comparison between cone/iris-based and InCise MLC-based CyberKnife plans for single and multiple brain metastases. J Appl Clin Med Phys 2016;17:184–99. 27685124 10.1120/jacmp.v17i5.6260 PMC5874093

[10] Murai T, Hattori Y, Sugie C et al. Comparison of multileaf collimator and conventional circular collimator systems in Cyberknife stereotactic radiotherapy. J Radiat Res 2017;58:693–700. 28199669 10.1093/jrr/rrw130 PMC5737677

[11] Ceylan C, Kucuk N, Bas Ayata H et al. Dosimetric and physical comparison of IMRT and CyberKnife plans in the treatment of localized prostate cancer. Rep Pract Oncol Radiother 2010;15:181–9. 24376947 10.1016/j.rpor.2010.10.003 PMC3863154

[12] Hossain S, Xia P, Huang K et al. Dose gradient near target-normal structure interface for nonisocentric CyberKnife and isocentric intensity-modulated body radiotherapy for prostate cancer. Int J Radiat Oncol Biol Phys 2010;78:58–63. 20133073 10.1016/j.ijrobp.2009.07.1752

[13] Macdougall ND, Dean C, Muirhead R. Stereotactic body radiotherapy in prostate cancer: Is rapidarc a better solution than cyberknife? Clin Oncol (R Coll Radiol) 2014;26:4–9. 24071450 10.1016/j.clon.2013.08.008

[14] Seppala J, Suilamo S, Tenhunen M et al. Dosimetric comparison and evaluation of 4 stereotactic body radiotherapy techniques for the treatment of prostate cancer. Technol Cancer Res Treat 2017;16:238–45. 28279147 10.1177/1533034616682156 PMC5616037

[15] Scobioala S, Kittel C, Elsayad K et al. A treatment planning study comparing IMRT techniques and cyber knife for stereotactic body radiotherapy of low-risk prostate carcinoma. Radiat Oncol 2019;14:143. 31399115 10.1186/s13014-019-1353-6 PMC6689170

[16] Sumida I, Shiomi H, Oh RJ et al. An optimization algorithm of dose distribution using attraction-repulsion model (application to low-dose-rate interstitial brachytherapy). Int J Radiat Oncol Biol Phys 2004;59:1217–23. 15234058 10.1016/j.ijrobp.2004.02.061

[17] Sumida I, Shiomi H, Yoshioka Y et al. Optimization of dose distribution for HDR brachytherapy of the prostate using attraction-repulsion model. Int J Radiat Oncol Biol Phys 2006;64:643–9. 16289907 10.1016/j.ijrobp.2005.09.008

[18] Kurosu K, Sumida I, Shiomi H et al. A robust measurement point for dose verification in delivery quality assurance for a robotic radiosurgery system. J Radiat Res 2017;58:378–85. 27811201 10.1093/jrr/rrw103 PMC5440860

[19] Low DA, Harms WB, Mutic S et al. A technique for the quantitative evaluation of dose distributions. Med Phys 1998;25:656–61. 9608475 10.1118/1.598248

[20] Niemierko A. A generalized concept of equivalent uniform dose (EUD). Med Phys 1999;26:1100 (Abstract). 10.1118/1.598063 9029544

[21] Burman C, Kutcher GJ, Emami B et al. Fitting of normal tissue tolerance data to an analytic function. Int J Radiat Oncol Biol Phys 1991;21:123–35. 2032883 10.1016/0360-3016(91)90172-z

[22] ICRU. Prescribing, recording, and reporting photon-beam intensity-modulated radiation therapy (IMRT). Journal of the ICRU 2010;10.

[23] Otto K. Volumetric modulated arc therapy: IMRT in a single gantry arc. Med Phys 2008;35:310–7. 18293586 10.1118/1.2818738

[24] Verbakel WF, Senan S, Cuijpers JP et al. Rapid delivery of stereotactic radiotherapy for peripheral lung tumors using volumetric intensity-modulated arcs. Radiother Oncol 2009;93:122–4. 19552979 10.1016/j.radonc.2009.05.020

[25] Kearney V, Descovich M, Sudhyadhom A et al. A continuous arc delivery optimization algorithm for CyberKnife m6. Med Phys 2018;45:3861–70. 10.1002/mp.13022 29855038

[26] Tomida M, Kamomae T, Suzuki J et al. Clinical usefulness of MLCs in robotic radiosurgery systems for prostate SBRT. J Appl Clin Med Phys 2017;18:124–33. 28691256 10.1002/acm2.12128 PMC5875821

[27] Akino Y, Shiomi H, Sumida I et al. Impacts of respiratory phase shifts on motion-tracking accuracy of the CyberKnife synchrony respiratory tracking system. Med Phys 2019;46:3757–66. 30943311 10.1002/mp.13523

