# Supplementary figures and images for: Development of raster scanning IMRT using a robotic radiosurgery system
Source: J Radiat Res. 2021 Jan 18;62(2):364–73. doi: 10.1093/jrr/rraa136 (PMC7948854; doi:10.1093/jrr/rraa136)

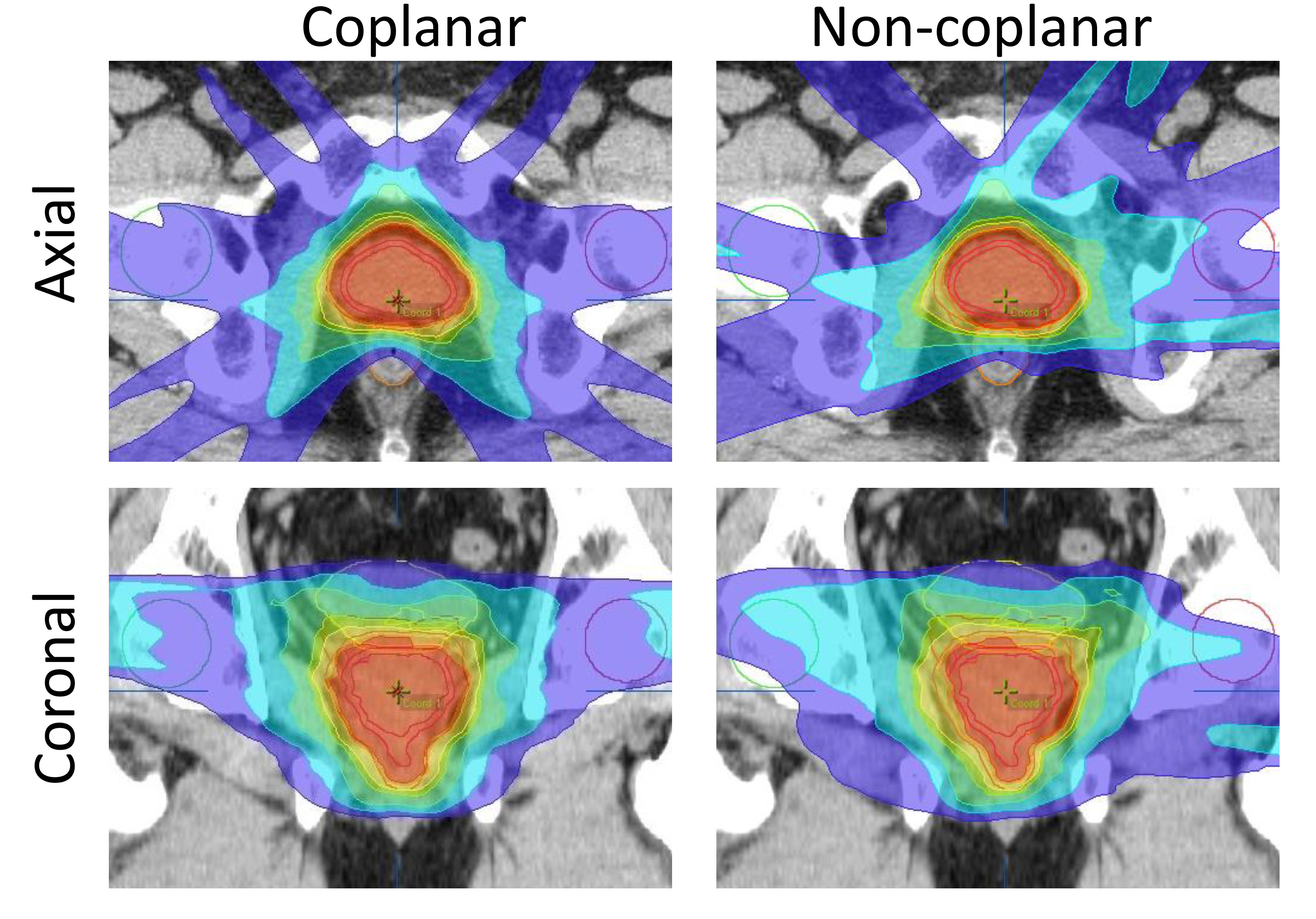

Supplement: Suppl_Figure_1_R5_rraa136 [file suppl_figure_1_r5_rraa136.zip › Suppl Figure 1_R5.tif]

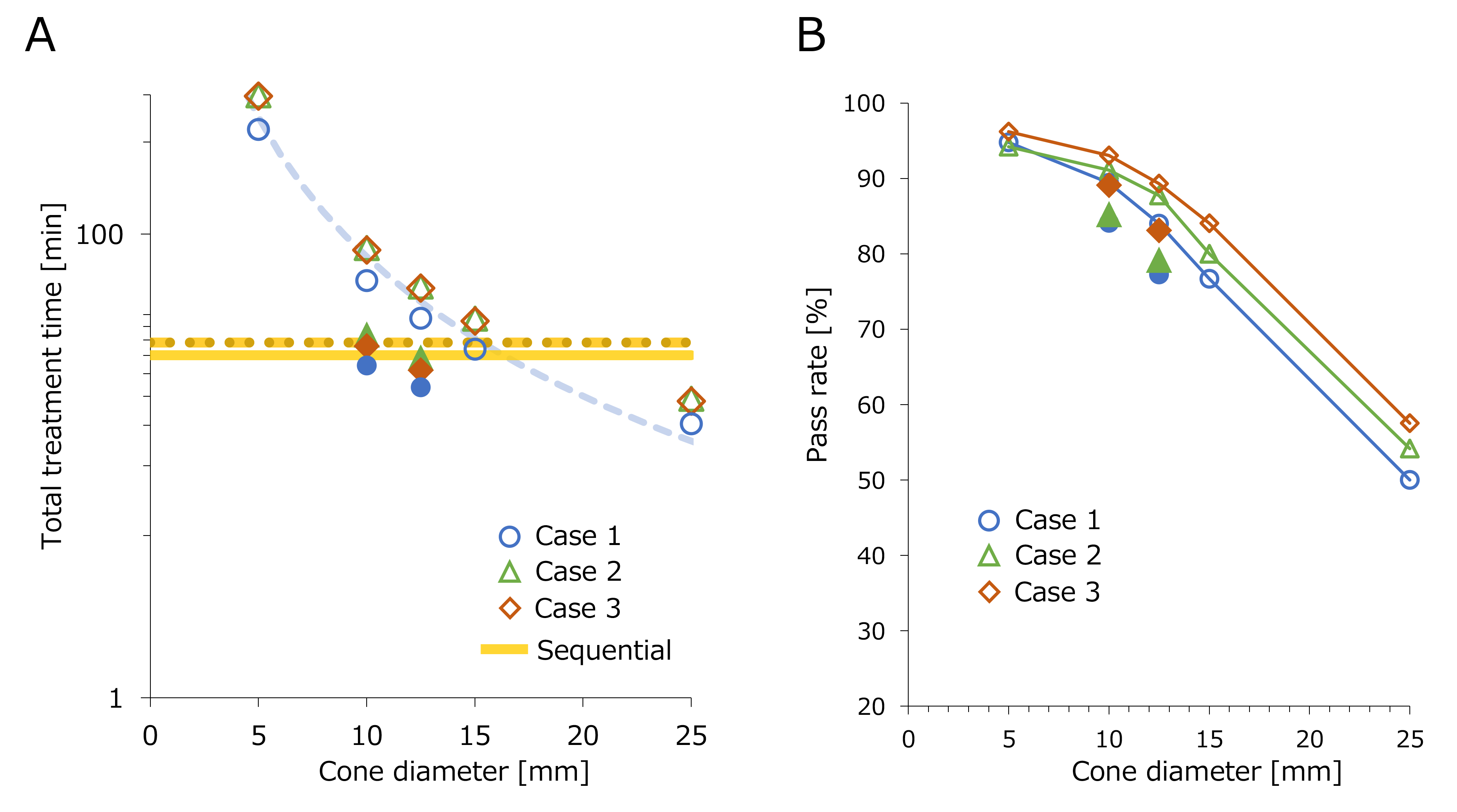

Supplement: Suppl_Figure_2_R5_rraa136 [file suppl_figure_2_r5_rraa136.png]

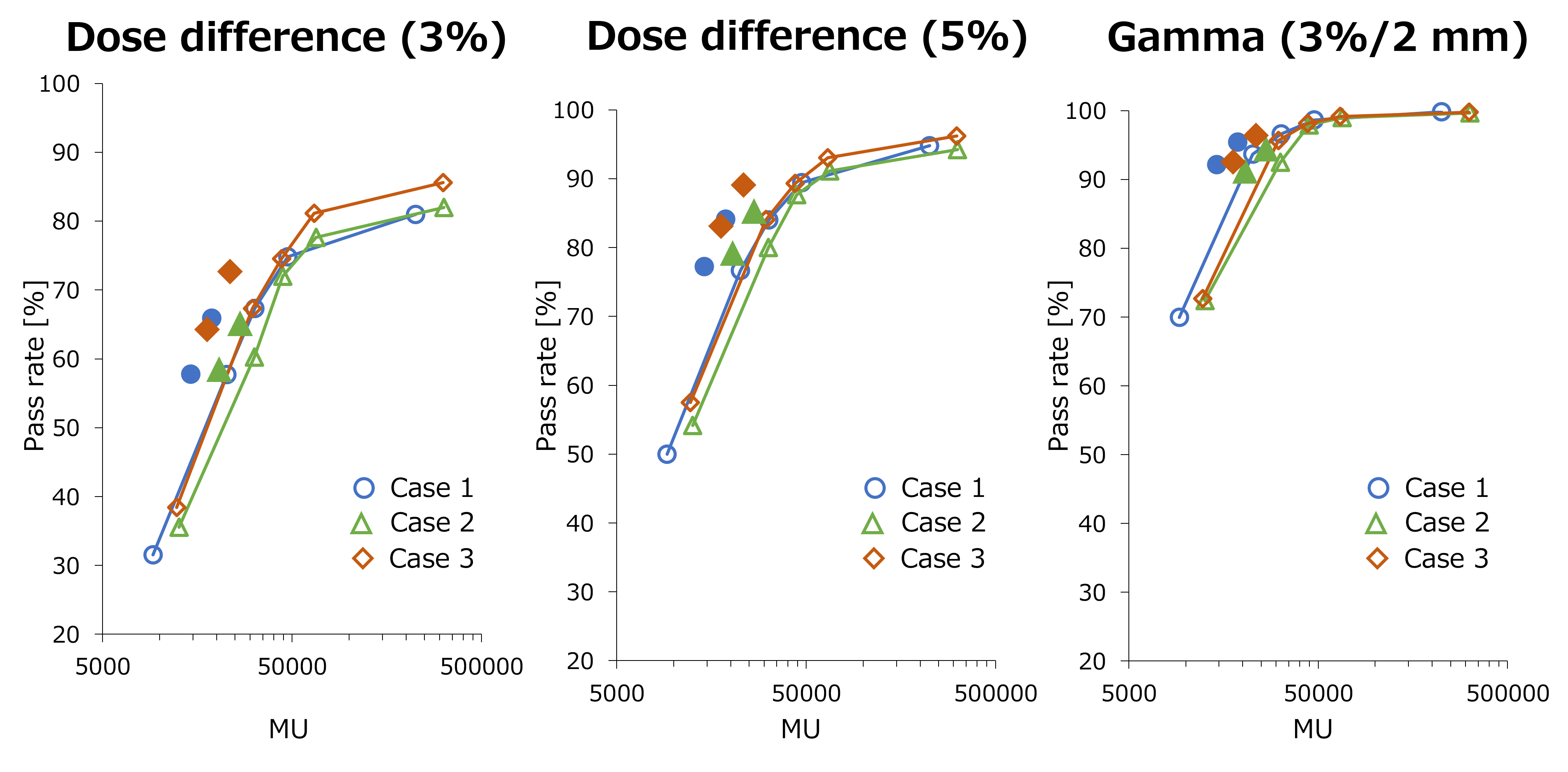

Supplement: Suppl_Figure_3_R5_rraa136 [file suppl_figure_3_r5_rraa136.png]
