# Supplementary material for: Development of raster scanning IMRT using a robotic radiosurgery system
Source: J Radiat Res. 2021 Jan 18;62(2):364–73. doi: 10.1093/jrr/rraa136 (PMC7948854; doi:10.1093/jrr/rraa136)
Supplement: Supplementary_Tables_R4_rraa136 [file supplementary_tables_r4_rraa136.docx]

Supplementary Table 1. Volume (cm^3^) of target, critical organs and the overlapped regions.

| Structure | Case #1 | Case #2 | Case #3 |
| --- | --- | --- | --- |
| PTV | 93.3 | 63.9 | 122.6 |
| Bladder | 91.7 | 105.4 | 151.5 |
| Rectum | 45.9 | 33.1 | 103.3 |
| Overlapped region |  |  |  |
| PTV ∩ Bladder | 2.6 | 8.4 | 20.1 |
| PTV ∩ Rectum | 2.4 | 0.9 | 5.4 |

Supplementary Table 2. Example of optimization parameters for CyberKnife sequential plans.

| Structure | Lower limit [cGy] | Upper limit [cGy] |  | Margin of shells [mm] | | |
| --- | --- | --- | --- | --- | --- | --- |
|  |  |  |  | Source | Posterior | Other |
| PTV | 3625 | 4000 |  |  |  |  |
| Shell 1 | - | 3600 |  | PTV | 3 | 3 |
| Shell 2 | - | 3300 |  | PTV | 4 | 7 |
| Shell 3 | - | 2900 |  | PTV | 8 | 12 |
| Shell 4 | - | 2000 |  | PTV | 15 | 20 |
| Shell 5 | - | 1200 |  | PTV | 35 | 35 |

Abbreviations: PTV, planning target volume.

Supplementary Table 3. The beam on time and treatment time of each plan

|  | Cone diameter | Raster scanning | | | | | | |  | Hybrid scanning | | Sequential | Original |
| --- | --- | --- | --- | --- | --- | --- | --- | --- | --- | --- | --- | --- | --- |
|  |  | 5 mm | 7.5 mm | 10 mm | 12.5 mm | 15 mm | 20 mm | 25 mm |  | 10 mm | 12.5 mm |  |  |
| #1 | MU | 212,306 | 80,310 | 46,379 | 31,699 | 22,384 | 13,721 | 9,502 |  | 19,397 | 14,368 | 4178 | 3,395 |
|  | Beam-on time [min] | 265.4 | 100.4 | 58.0 | 39.6 | 28.0 | 17.2 | 11.9 |  | 24.2 | 18.0 | 5.2 | 4.2 |
|  | Estimated treatment time [min] | 269.1 | 104.1 | 61.7 | 43.3 | 31.7 | 20.9 | 15.6 |  | 28.0 | 21.7 | 30.0 | - |
|  |  |  |  |  |  |  |  |  |  |  |  |  |  |
| #2 | MU | 310,499 | 114,167 | 65,615 | 43,816 | 30,991 | 18,184 | 12,388 |  | 25,659 | 19,856 | 3978 | 3,238 |
|  | Beam-on time [min] | 388.1 | 142.7 | 82.0 | 54.8 | 38.7 | 22.7 | 15.5 |  | 32.1 | 24.8 | 5.0 | 4.0 |
|  | Estimated treatment time [min] | 391.8 | 146.4 | 85.7 | 58.5 | 42.4 | 26.4 | 19.2 |  | 35.8 | 28.6 | 34.0 | - |
|  |  |  |  |  |  |  |  |  |  |  |  |  |  |
| #3 | MU | 294,226 | 114,498 | 65,679 | 44,140 | 30,085 | 17,788 | 12,129 |  | 23,266 | 17,784 | 4000 | 3,568 |
|  | Beam-on time [min] | 367.8 | 143.1 | 82.1 | 55.2 | 37.6 | 22.2 | 15.2 |  | 29.1 | 22.2 | 5.0 | 4.5 |
|  | Estimated treatment time [min] | 371.5 | 146.8 | 85.8 | 58.9 | 41.3 | 25.9 | 18.9 |  | 32.8 | 26.0 | 34.0 | - |

Note: The estimated treatment time for raster scanning and hybrid scanning plans were calculated with consideration of arm motion time as 3.7 min for eight beams. Beam-on time of the original plans were calculated with 800 MU/min dose rate, although clinically used dose rate for prescription of 2.4 Gy/fraction is lower than 300 MU/min.

| Supplementary Table 4. The passing rate of dose difference calculated between the fluence maps calculated with raster scanning and Novalis plans with criteria of 5%. | | | | | | | | | | | |
| --- | --- | --- | --- | --- | --- | --- | --- | --- | --- | --- | --- |
| Case | Cone diameter | Raster scanning | | | | | | |  | Hybrid scanning | |
|  |  | 5 mm | 7.5 mm | 10 mm | 12.5 mm | 15 mm | 20 mm | 25 mm |  | 10 mm | 12.5 mm |
| #1 | Mean | 94.3 | 93.2 | 88.9 | 81.1 | 70.8 | 51.7 | 42.8 |  | 83.1 | 74.7 |
|  | Minimum | 89.1 | 88.0 | 83.8 | 75.2 | 61.4 | 41.9 | 31.9 |  | 78.5 | 67.9 |
|  | Maximum | 98.3 | 97.2 | 94.3 | 88.7 | 81.3 | 64.5 | 57.2 |  | 88.8 | 81.7 |
|  |  |  |  |  |  |  |  |  |  |  |  |
| #2 | Mean | 94.9 | 94.3 | 91.7 | 86.2 | 82.0 | 62.4 | 53.5 |  | 85.4 | 79.4 |
|  | Minimum | 90.9 | 90.3 | 88.1 | 81.2 | 75.6 | 52.5 | 41.9 |  | 78.1 | 70.3 |
|  | Maximum | 99.1 | 97.9 | 95.6 | 91.1 | 88.4 | 73.2 | 67.1 |  | 89.8 | 84.7 |
|  |  |  |  |  |  |  |  |  |  |  |  |
| #3 | Mean | 96.3 | 95.7 | 93.4 | 90.2 | 85.3 | 66.2 | 57.4 |  | 88.9 | 84.0 |
|  | Minimum | 92.7 | 92.4 | 89.3 | 83.3 | 78.5 | 58.0 | 47.8 |  | 85.4 | 80.7 |
|  | Maximum | 98.7 | 97.9 | 96.5 | 93.2 | 88.9 | 73.9 | 67.1 |  | 91.4 | 87.1 |
